# Association between education and blood lipid levels as income increases over a decade: a cohort study

**DOI:** 10.1186/s12889-018-5185-3

**Published:** 2018-02-27

**Authors:** Macarena Lara, Hugo Amigo

**Affiliations:** Department of Nutrition, University of Chile, Independencia 1027, 8380453 Santiago, Chile

**Keywords:** Education, Income change, Blood lipids, Health inequalities, Gender, Mediation analysis

## Abstract

**Background:**

Cardiovascular risk factors have increased along with economic development, but it is not clear if this tendency differs by education. The aim of this study was to analyze the effect of education on blood lipid levels while income increases over a decade in Chilean adults.

**Methods:**

A cohort study was conducted from 3092 births in Limache Hospital between 1974 and 1978, of which 998 people were randomly selected in 2000 and 650 followed up in 2010. Using mediation analysis, the controlled direct effect (CDE) of education in 2000 on blood lipid levels in 2010: triglycerides (TG), total cholesterol (TC), LDL cholesterol (LDL) and HDL cholesterol (HDL) while setting the mediator, income, to “increased” between 2000 and 2010 was estimated. The results were expressed through the CDE and its 95% confidence interval (CI).

**Results:**

Of the 650 adults, 24% had low education (≤ 8 years) and 60% increased their income. The mediation analysis showed that, when setting income to “increased”, women with low education had worse lipid profiles than women with high education: TG_CDE_ = 14 (CI = − 7;34), TC_CDE_ = 4 (CI = − 8;15), LDL_CDE_ = 1 (CI = − 8;9), HDL_CDE_ = − 3 (CI = − 7;0), while men with low education had better lipid profiles than men with high education: TG_CDE_ = − 2 (CI = − 41;38), TC_CDE_ = − 12 (CI = − 29;5), LDL_CDE_ = − 12 (CI = − 24;1), HDL_CDE_ = 1 (CI = − 5;6).

**Conclusion:**

Faced with a rise in income, there was a trend to associate low education with worse lipid profiles in women and better lipid profiles in men.

## Background

Currently, cardiovascular diseases are the main cause of death in Chile and worldwide [1–3]. In 2015, the World Health Organization reported that 17.7 millions of people died from cardiovascular diseases (equivalent to 31% of the total deaths in the world) and were responsible for 27% of the deaths in Chile [1, 3]. Projections indicate that the number of deaths due to cardiovascular diseases will increase to 23.3 million by 2030 [4].

Among the modifiable risk factors of cardiovascular diseases are smoking, obesity, sedentary lifestyle, hypertension, diabetes mellitus and dyslipidemia [5]. It has been observed that each of these factors has increased quickly in recent decades reaching high levels globally [6]. These trends are also present in Chile since, according to the last National Health Survey conducted between 2009 and 2010, 41% of Chilean adults smoke, 25% are obese, 89% are sedentary, 27% have hypertension, 9% diabetes and 39% high cholesterol [7].

It has been observed that the prevalence of cardiovascular risk factors has increased along with the economic improvements, especially in developing countries [2]. In Latin America, poverty has fallen significantly since 2000. The proportion of people living under poverty line in the region has been almost cut in half since 2000 to 23% in 2015 [8]. Chile has been one of Latin America’s fastest-growing economies in recent decades, reducing the poverty rate from 20% in 2000 to 15% a decade later [9] and becoming one of the most economically stable countries in South America [10, 11].

Parallel to this fast economic growth, cardiovascular risk factors rates increased in Chilean adults [7, 12]. Globalization, urbanization and industrialization have accompanied this economic transition, impacting population lifestyles and leading to higher rates of cardiovascular diseases [2, 13]. Currently, Chilean adults are living in an obesogenic enviroment with high rates of sedentarism and unhealthy diets, high in sugar, fat and salt, which all contribute to cardiovascular diseases [14, 15].

In the last National Health Survey, most of the cardiovascular risk factors were more frequent at lower education level, showing a gradient toward improved health outcomes as education increases [7]. It has been reported that people with low education are more susceptible to acquiring less healthy eating patterns and lifestyles, and this could be accentuated when their income increases without an improvement in their education [16, 17].

Three mechanisms have been proposed to explain the relationship between education and health [17]. The first is that education increases knowledge and health behaviors directly [18, 19] which can be seen in studies that show an association between education and consumption of healthier food as well as increased physical activity [20, 21]. The second possible mechanism is that increased education can lead to better employment opportunities that allow people to live in better, less stressful environments with a higher availability of health food options and recreational areas [22, 23]. The last mechanism is that higher education is associated with psychosocial factors such as a sense of control, social position and social support, that help to reduce barriers to change and maintain improvements in health behaviors [24–26].

Considering the economic improvements, it is still unclear whether there are differences in adults blood lipid levels by education status. This information could be useful to understand the mechanisms involved in the production of this type of health inequalities. Therefore, the objective of this study was to analyze the effect of education on blood lipid levels while considering the change in income between 2000 and 2010 in adults born between 1974 and 1978 in Limache Hospital, Chile.

## Methods

### Study population and design

A concurrent cohort study was undertaken in Limache and Olmué, which are adjoining towns located 108 km from Santiago, the capital of Chile. Both are considered semi-rural communities, and their main economic activity is agriculture and more recently tourism. In 2000, 998 adults were randomly selected from a sampling frame of 3092 newborns registered between 1974 and 1978 in Limache Hospital [27]. In 2010, 650 of them were followed-up.

### Collection of information

Socio-demographic information was collected using a structured questionnaire that asked about age, years of education at baseline and per-capita income in 2000 and 2010. Salaries, pensions, social program benefits and dividends were all included as sources of income. Per-capita income was calculated from the monthly household income divided by the number of household members weighted by the OECD equivalence scale [28] and corrected for inflation during the study period [29]. Socioeconomic level in 2000 was described using a validated index that consisted of a two-dimensional social grade matrix that combined the educational level and occupation of the head of the household and categorized the socioeconomic level into five strata: high, middle-high, middle, middle-low and low [30]. As very few participants were observed in the highest two socioeconomic levels, they were merged into the middle group.

Weight and height in 2010 were measured to calculate body mass index (BMI). Bicipital, tricipital, subscapular and suprailiac skinfold thickness were measured using a Harpenden Caliper to compute skinfold thickness sum in 2010. All these measurements were taken by standardized and periodically monitored nutritionists, using as reference the protocol for anthropometric measures proposed by the World Health Organization [31].

Physical activity in 2010 was measured using the International Physical Activity Questionnaire (IPAQ), which has been translated and validated in different countries [32]. Energy intake in 2010 was estimated with a quantified food frequency questionnaire applied by nutritionists who collected information on food intake during the last 30 days of each participant by interview.

Measurements and blood specimens (with 12 h fasting) were obtained in a hospital or local health facility. Trained and supervised nurses measured blood lipid levels. Blood samples were processed at Limache Hospital and frozen for later laboratory analysis. Blood lipid levels were measured by enzymatic colorimetric method: tryglicerides (HUMAN Ref 10,724), total cholesterol (HUMAN Ref 10,028), HDL cholesterol (HUMAN Ref 10,084) and LDL cholesterol (HUMAN Ref 10,094). In adults with tryglicerides levels below 400 mg/dL, LDL cholesterol level was calculated using the Friedewald formula [33].

Information about maternal education and family history of obesity and dyslipidemia was collected via questionnaire. Birth weight was collected from the official hospital register.

### Analysis

Education was dichotomized into “low education” if the person had 8 or less years of education approved and “high education” if the person had more than 8 years approved. The income change was dichotomized into “increases” if income increased by 10% or more between 2000 and 2010 and “decreases” if not. The outcomes were continuous blood lipid levels in 2010 (triglycerides, total cholesterol, HDL and LDL cholesterol).

Mediation analysis was used [34] considering “education in 2000” as exposure, "income change between 2000 and 2010" as the mediator and “blood lipid levels in 2010” as outcomes. This analysis is based on a causal mediation model and estimates the controlled direct effect (CDE). The CDE corresponds to the effect of exposure (education in 2000) on the outcome in 2010 when the mediator (income change between 2000 and 2010) is set to 1 (income increased). In other words, it is the effect of all paths from exposure to outcome that do not pass through the mediator.

Separate models for each outcome were fit for the total sample and by sex, the results were expressed through the CDE and 95% confidence intervals (CI) and prior to analysis it was tested whether there was interaction between exposure and a mediator, considering a *p*-value < 0.10 to be significant. Confounding variables such as sex, age, birth weight, income at baseline, maternal education and family history of obesity and dyslipidemia were considered. Losses to follow up were quantified and the characteristics of the group that remained in the study were contrasted with the group that was lost to verify whether the losses to follow up were related to exposure and outcome which could introduce a selection bias [35]. Inverse probability of censoring weighting was used to solve this problem [35, 36]. Raw models and models adjusted for confounders and weighted by inverse probability of censoring to consider losses to follow up were presented.

## Results

From 998 adults at baseline, 35% were loss to follow up during the ten years of the study. Therefore, 650 participants were measured in both 2000 and 2010.

Table 1 describes the characteristics of the cohort in 2000 and 2010. At baseline, the age of the sample ranged between 22 and 28 years, the mean of education level was below a high school degree and the monthly per-capita income was 97 USD, 19% higher in men than women (*p* = 0.005). Distribution of socioeconomic level was different by sex, the proportion of low socioeconomic level was almost the double in women than men. Nearly a quarter of the adults had low education and the difference in this proportion was not significant between women and men (*p* = 0.326). Between 2000 and 2010, 60% of the sample increased greater than 10% their per-capita income.

**Table 1:** Description of the cohort at baseline (2000) and follow-up (2010)

	Total	Women	Men	
	(*n* = 650)	(*n* = 428)	(*n* = 222)	
Characteristics in 2000	Mean ± SD	Mean ± SD	Mean ± SD	*p*
Age (years)	25 ± 2	25 ± 2	25 ± 2	0.333
Education (years approved)	11 ± 3	11 ± 3	11 ± 3	0.118
Per-capita income (USD/month)	97 ± 90	89 ± 82	111 ± 103	0.005
	%	%	%	*p* ^a^
Socioeconomic level				
low	28	34	18	< 0.001
middle low	38	29	57	
middle and high	34	37	25	
Low Education (≤ 8 years)	24	23	26	0.326
Increased income (2000–2010)	60	58	62	0.416
Characteristics in 2010	Mean ± SD	Mean ± SD	Mean ± SD	*p*
Triglycerides (mg/dL)	133 ± 73	123 ± 66	152 ± 81	< 0.001
Total cholesterol (mg/dL)	182 ± 36	182 ± 36	183 ± 36	0.871
LDL cholesterol (mg/dL)	111 ± 27	112 ± 27	108 ± 27	0.075
HDL cholesterol (mg/dL)	48 ± 12	47 ± 12	48 ± 12	0.458
Glycemia (mg/dL)	93 ± 18	92 ± 18	95 ± 17	0.077
Systolic blood pressure (mm Hg)	119 ± 16	116 ± 15	125 ± 15	< 0.001
Diastolic blood pressure (mm Hg)	74 ± 12	73 ± 11	76 ± 12	< 0.001
BMI (kg/m^2^)	29 ± 5	29 ± 5	28 ± 4	0.003
Skinfold thickness sum (mm)	76 ± 27	85 ± 24	59 ± 22	< 0.001
Physical activity (METs/week)	3486 ± 3920	2536 ± 2955	5317 ± 4809	< 0.001
Energy intake (Kcal/day)	2285 ± 920	1946 ± 688	2948 ± 956	< 0.001
	%	%	%	*p* ^a^
Triglycerides > 150 mg/dL	33	28	43	< 0.001
Total cholesterol > 200 mg/dL	28	27	31	0.286
LDL cholesterol > 130 mg/dL	21	22	21	0.766
HDL cholesterol < 40 mg/dL	28	28	27	0.737
Glycemia > 100 mg/dL	21	18	26	0.027
Blood Pressure ≥ 140/90 mmHg	15	12	20	0.004
Nutritional status				0.022
normal	25	24	28	
overweight	41	39	45	
obesity	34	37	26	

Student t-test; ^a^ Chi^2^ test

In 2010, the blood lipid profiles were similar by sex with the exception of triglycerides, which were 29 mg/dL higher in men than women (*p* <  0.001). Men also had an 8% (*p* = 0.027) higher prevalence of hyperglycemia and the proportion of men with high blood pressure was almost the double that in women (*p* = 0.004). On the other hand, women had 1 kg/m^2^ higher BMI and 26 mm higher skinfold thickness sum than men (*p* <  0.004). Although women reported having a lower daily energy intake, their physical activity was much lower than men (*p* <  0.001). It is important to mention that 75% of the adults were overweight or obese at 35 years old. While overweight was more frequent in men obesity was more frequent in women (Table 1).

Table 2 shows the differences in blood lipid levels and other related variables by education in the total sample and separated by sex. Mean blood lipid levels tended to be worse in women and better in men with low education in comparison to their peers with high education. Also, women with low education had 2 kg/m^2^ higher BMI and 8 mm higher skinfold thickness sum than women with high education, while men with low education had 2334 METs/week more and 14 mm less of skinfold thickness sum than men with high education.

**Table 2:** Differences in blood lipid levels and related variables by education level

Variables in 2010	Total			Women			Men		
	Low education	High education	*p*	Low education	High education	*p*	Low education	High education	*p*
Triglycerides (mg/dL)	136 ± 6	132 ± 3	0.548	133 ± 6	120 ± 4	0.098	142 ± 11	156 ± 6	0.246
Total cholesterol (mg/dL)	183 ± 3	182 ± 2	0.667	186 ± 4	181 ± 2	0.217	179 ± 5	184 ± 3	0.345
LDL cholesterol (mg/dL)	110 ± 2	111 ± 1	0.745	114 ± 3	112 ± 1	0.423	104 ± 3	110 ± 2	0.133
HDL cholesterol (mg/dL)	47 ± 1	48 ± 1	0.499	46 ± 1	48 ± 1	0.090	50 ± 2	48 ± 1	0.266
Physical activity (METs/week)	4117 ± 4428	3288 ± 3730	0.021	2369 ± 2773	2584 ± 3009	0.529	7041 ± 5110	4707 ± 4560	0.001
Energy intake (Kcal/day)	2321 ± 1006	2273 ± 893	0.578	1830 ± 631	1980 ± 701	0.062	3154 ± 978	2876 ± 941	0.061
BMI (kg/m^2^)	29 ± 6	28 ± 5	0.086	30 ± 6	28 ± 5	0.002	27 ± 5	28 ± 4	0.119
Skinfold thickness sum (mm)	75 ± 32	76 ± 25	0.523	91 ± 26	83 ± 24	0.007	48 ± 22	62 ± 21	< 0.001

Values expressed as mean ± standard deviation; Student t-test; Low education: ≤ 8 years, High education > 8 years

Before the mediation analysis was performed, interaction between exposure (education in 2000) and mediator (income change between 2000 and 2010) was confirmed when the effect on HDL cholesterol (*p* = 0.052) was analyzed. It was also necessary to use inverse probability of censoring weighting to avoid selection bias after observing that more men, people with higher education and less obesity were lost.

Table 3 shows the results of the mediation analysis that measured the effect of education in 2000 on blood lipid levels in 2010 had income increased during the decade studied. Crude and adjusted models are presented weighted by the inverse probability of censoring. As mentioned previously, the results for each type of analysis were expressed as the CDE and its CI for the total sample and by sex (Table 3).

**Table 3:** Controlled direct effect (CDE) of education in 2000 on blood lipid levels in 2010 when income change is set to “increased”

Variables In 2010	Crude Model			Adjusted and weighted model ^a^ CDE (95% CI)		
	CDE (95% CI)					
	Total	Women	Men	Total	Women	Men
Triglycerides (mg/dL)	11 (−6; 30)	18 (−1; 39)	2 (− 34; 40)	9 (− 11; 28)	14 (− 7; 34)	-2 (− 41; 38)
Total cholesterol (mg/dL)	-2 (− 10; 7)	3 (− 7; 14)	− 12 (− 25; 2)	−3 (− 12; 7)	4 (− 8; 15)	−12 (− 29; 5)
LDL cholesterol (mg/dL)	−4 (− 10; 3)	1 (− 7; 9)	−13 (− 23; − 3)	−4 (− 11; 3)	1 (−8; 9)	− 12 (− 24; 1)
HDL cholesterol (mg/dL)	−2 (− 5; 0)	−4 (− 7; − 1)	1 (− 4; 6)	− 2 (− 5; 1)	− 3 (− 7; 0)	1 (−5; 6)

^a^Models adjusted for sex, age, birth weight, income at baseline, maternal education and family history of dyslipidemia

Models adjusted for confounders and weighted for loss to follow-up showed that setting income to “increased” between 2000 and 2010, women with low education had a non-significant trend toward worse lipids profiles than their peers with high education: CDE_TG_ = 14 (CI = − 7; 34), CDE_CT_ = 4 (CI = − 8; 15), CDE_LDL_ = 1 (CI = − 8; 9), CDE_HDL_ = − 3 (CI = − 7; 0). In contrast, men with low education had a non-significant trend toward better lipid profiles than men with high education: CDE_TG_ = − 2 (CI = − 41; 38), CDE_CT_ = − 12 (CI = − 29; 5), CDE_LDL_ = − 12 (CI = − 24, 1), CDE_HDL_ = 1 (CI = − 5, 6) (Table 3).

## Discussion

Faced with an increase in income between 2000 and 2010, women with low education had a trend toward worse lipid profiles while men with low education had a trend toward better lipid profiles, compared to their peers with high education.

The mean of years of education in the adults studied was one year lower than the mean reported for the Chilean population between 22 and 28 years in 2000, and while the proportion of adults with primary degree or less was almost a quarter of the sample studied it was 18% nationally, according to the National Socioeconomic Characterization Survey conducted in 2000 [37].

The improvement in per-capita income was consistent with the economic growth in this period at the national level [9, 10]. More than the half of the participants in this study increased their monthly per-capita income, which could be attributed to the change in the main economic activity in the place studied. While agriculture was most important in 2000, it was displaced by social community services, such as tourism, education, health, among others, which ranked first in 2009 [37, 38].

The mean of cardiometabolic and anthropometric parameters were similar to the national average of the same age group [39]. Men had a higher frequency of elevated tryglicerides, hyperglycemia and hypertension than women, as has been previously reported for young adults [40, 41]. The fact that three quarters of the cohort reported being overweight or obese, and that this prevalence is similar to the Chilean population, demonstrates that the overweight and obesity epidemic is an important public health issue in Chile [7] as is the case with most countries internationally [42].

Women with low education had higher BMI and skinfold thickness sum than women with high education. This is consistent with other studies that have observed higher anthropometric measurements in less educated women as a consequence of genetic and environmental factors interacting [43–46]. Women did not have difference in physical activity by education and this could be explained because most women sampled had sedentary occupations regardless their education status. Instead men with low education had higher physical activity and lower skinfold thickness sum, possibly because their occupation required more physical work and this could have reduced body fat and could have improved their blood lipid levels. There was no observed difference in BMI by education in men and it could be possibly because it is an anthropometric index that which is not able to differentiate lean from fat mass. The daily energy intake in both women and men was slightly higher than the reported by the National Food Survey conducted in Chile in 2009–2010 [47]. The lack of difference in daily energy intake by education could be explained by the recall and social desirability bias associated with the instrument [48–50].

Low education was associated with worse lipid profiles in women and better lipid profiles in men (both without statistical significance, but with a tendency toward these results). The lack of statistical significance could be interpreted in two ways: there is possibly a significant effect, but it would be necessary to increase the sample size or work with a more heterogeneous sample because socioeconomic homogeneity of the sample could be masking relevant effects of education on blood lipid levels and in a more heterogeneous scenario they could be visible. The other interpretation is that education is really not directly influencing the current blood lipid levels and other variables may have more to do with them. Another possibility is that the effect of education on blood lipid levels is small because the biological variability coefficient of these blood parameters is not high enough to display a large magnitude of the effect, especially for total cholesterol, LDL and HDL, whose percentages of intra-individual biological variability do not exceed 9% [51]. The concentrations of blood triglycerides may be less stable and more easily affected by recent meals, this being the reason that 12 h of fasting were required before the blood sample was drawn [51, 52].

It is important to mention that this is one of the first studies using a cohort study that investigates the effect of education on blood lipid levels in adults aged 35 years, considering an improvement in income over the past decade, using a mediation analysis that included exposure-mediator interaction and considering the technique of “inverse probability weighting” to correct for selection bias.

Another point to note is that according to the records of the Demography of the National Institute of Statistics of Chile, births at Limache Hospital who formed the sampling frame of this study accounted for 99% of live births in the communes of Limache and Olmué between 1974 and 1978. Furthermore, the quantification of blood lipid levels was performed in an internationally certified laboratory, in order to reduce analytical variability and ensure the delivery of accurate measurements and accurate blood parameters values.

Among the weaknesses of this study is the difficulty of measuring the mediator: “per-capita income”, because generally people tend to overestimate or underestimate family income depending on the context in which the interview is conducted [53]. To minimize this information bias, we worked with trained professionals and supervised who applied the socioeconomic survey at the home of each participant and spent all the time needed to collect and accurately as possible socioeconomic information in the most complete way. Also, while we used inverse probability weighting to correct for selection bias with regard to observed variables, if those lost to follow-up were different with regard to unmeasured variables, some selection bias may remain.

For future research, it would be interesting to continue to follow participants over time to assess whether the effects change with age. The cohort could also be opened to other communities thereby increasing heterogeneity and sample size. In turn, studying the effect of characteristics at birth on blood lipid levels in adulthood would complement the results and would provide relevant information, since literature has raised the need for cohort studies with sufficient power and statistical data to generate robust and conclusive evidence in this area [54, 55].

## Conclusion

This study found a qualitative interaction by sex in the effect of education on blood lipid levels when income increased over a decade: low education was associated with worse lipid profiles in women and with better lipid profiles in men. This information could help to better understand the pathways involved in the generation of this kind of socioeconomic inequalities. It also suggests that public health policy in Chile or Latin America that aims to reduce and/or control blood lipid levels in adults should consider men and women differently in order to be more effective.

## Abbreviations

BL: Blood lipid levels
BMI: Body mass index
CDE: Controlled direct effect
CI: 95% confidence interval
HDL: HDL cholesterol
IPAQ: International Physical Activity Questionnaire
LDL: LDL cholesterol
TC: Total cholesterol
TG: Triglycerides
